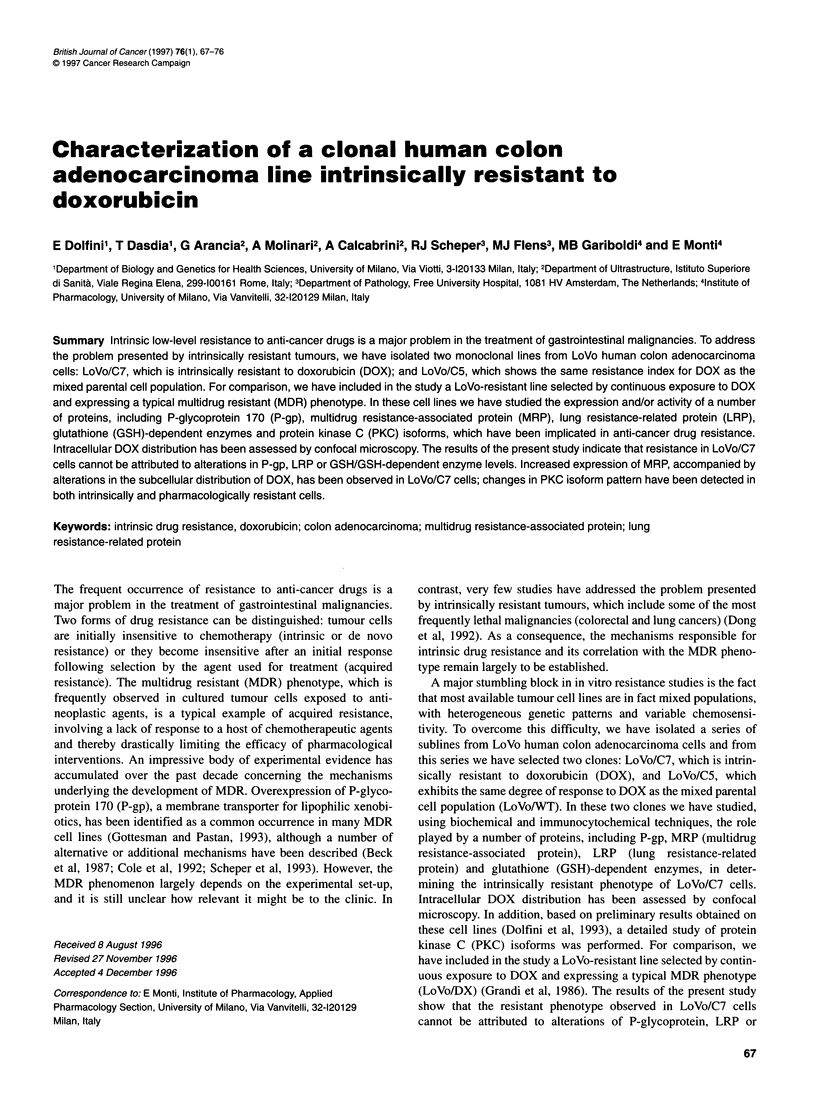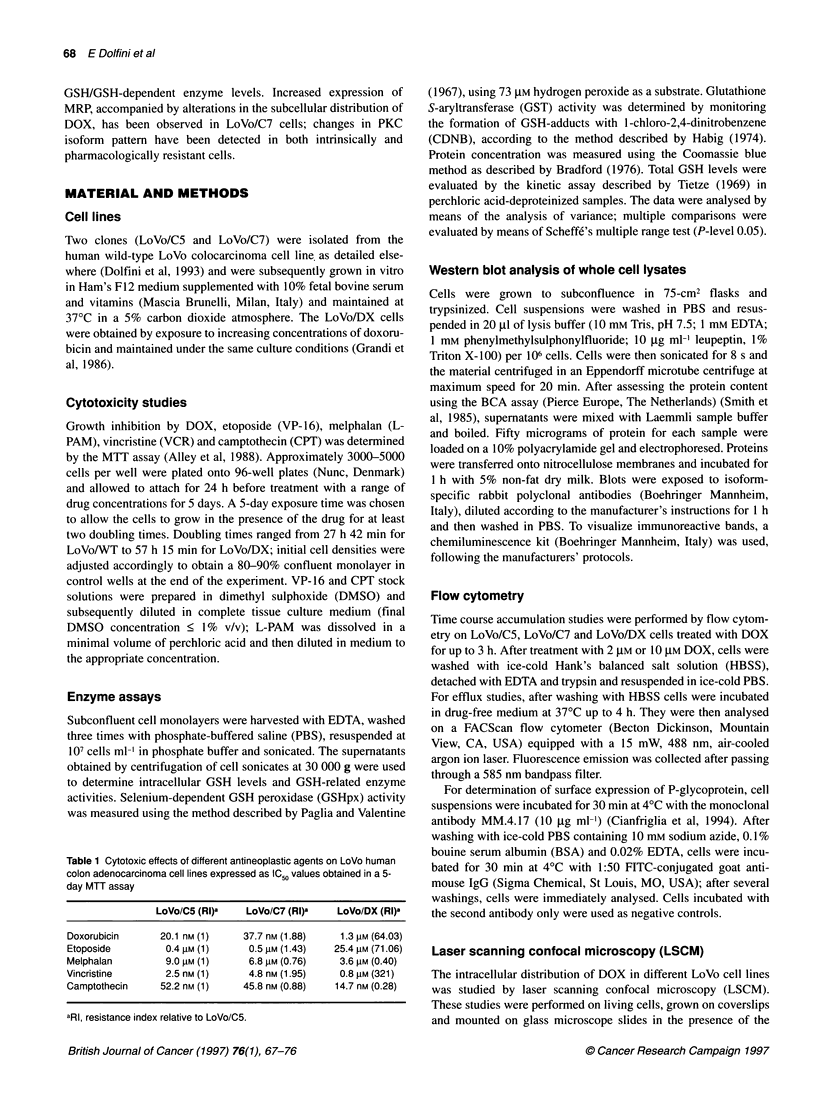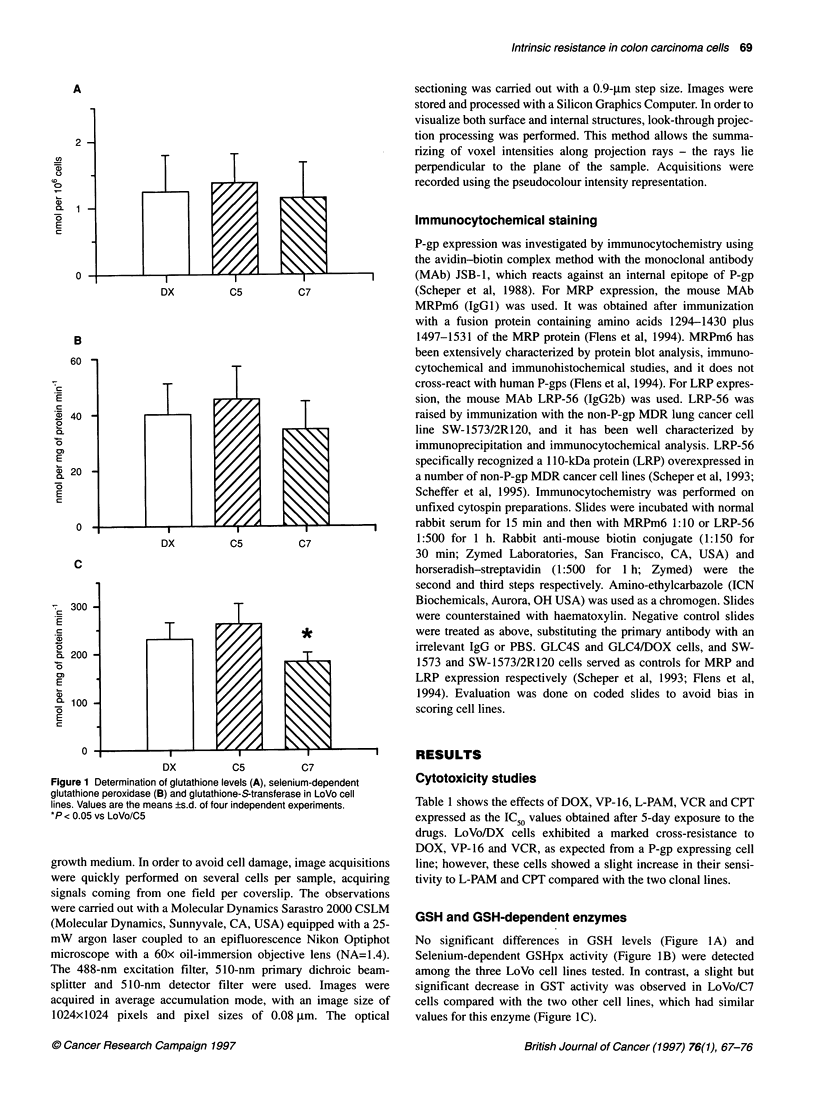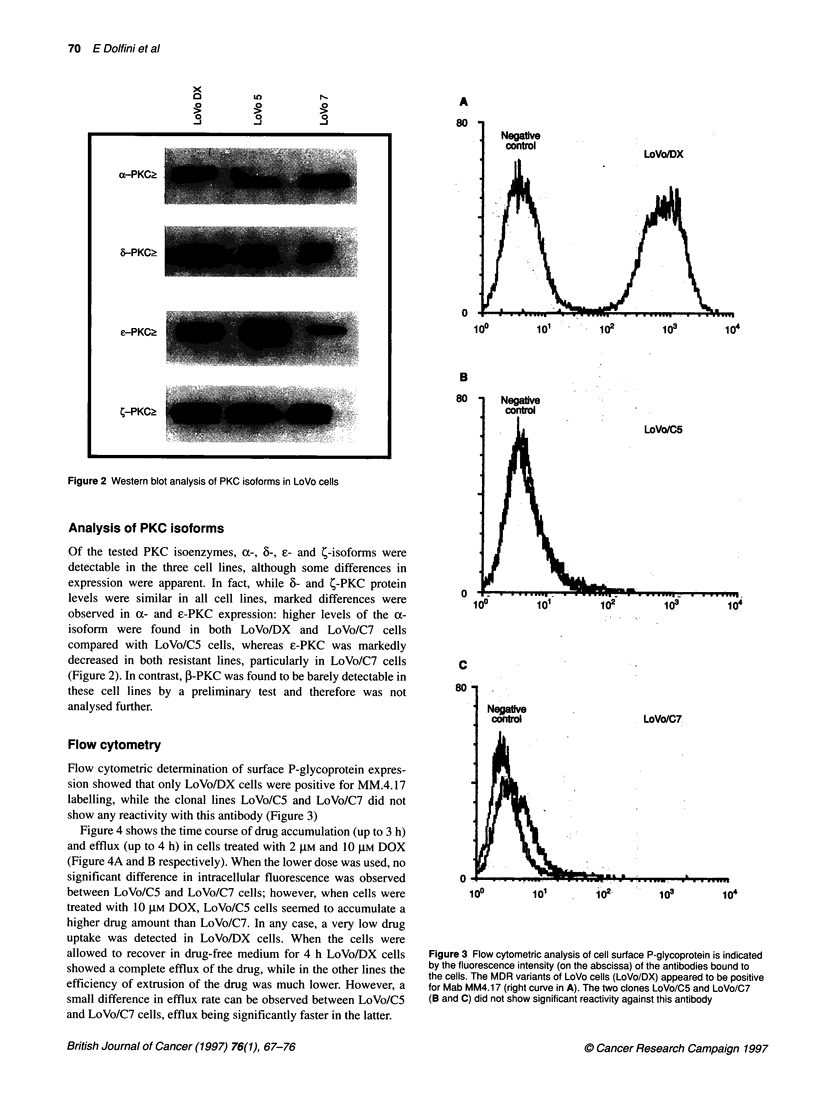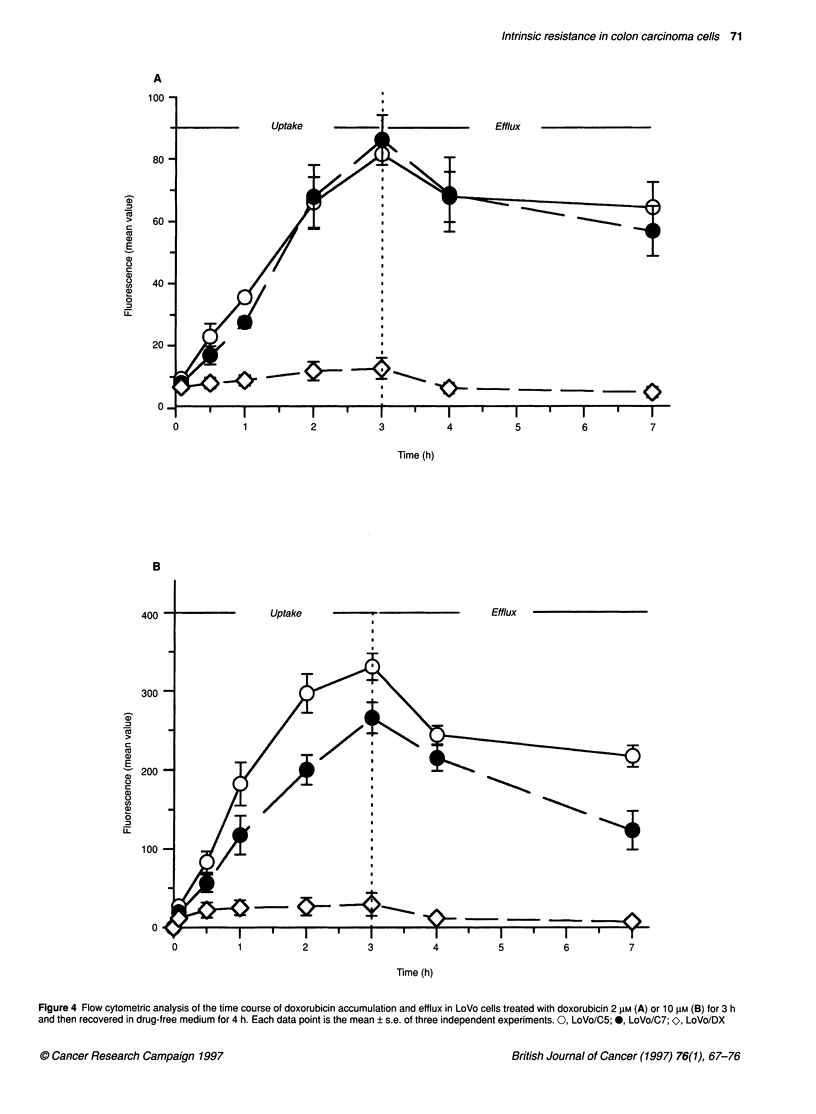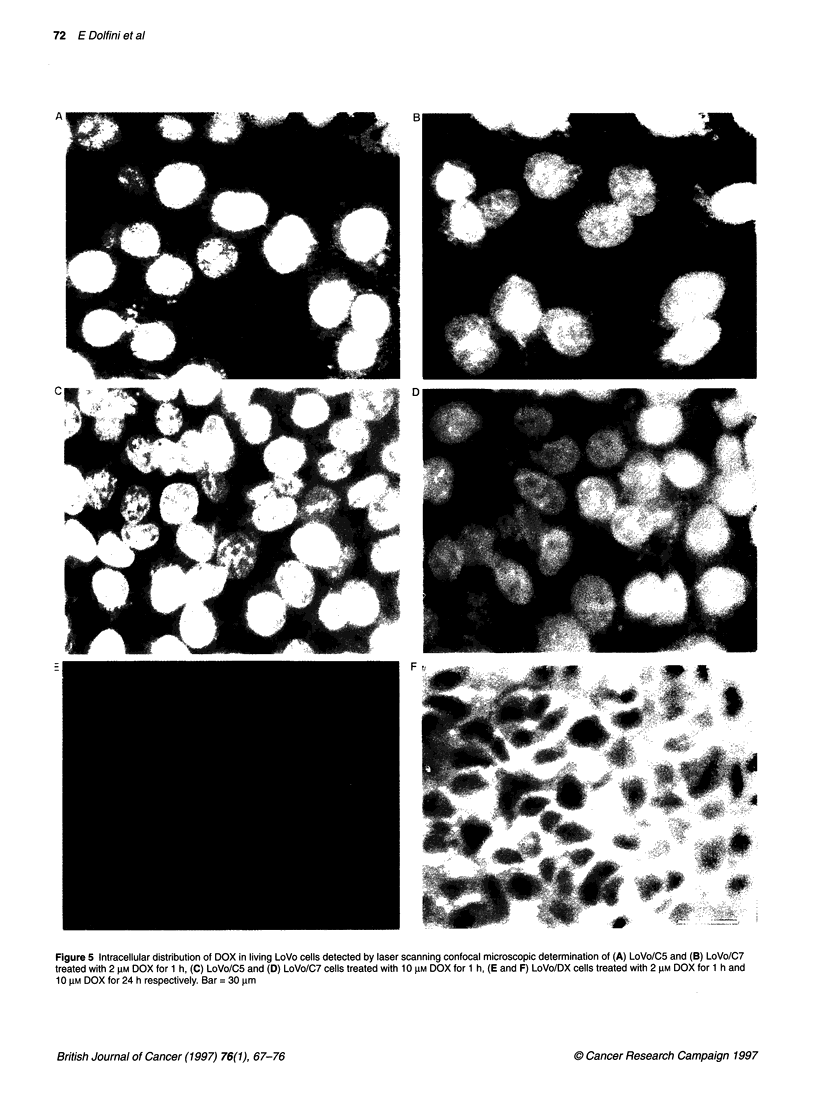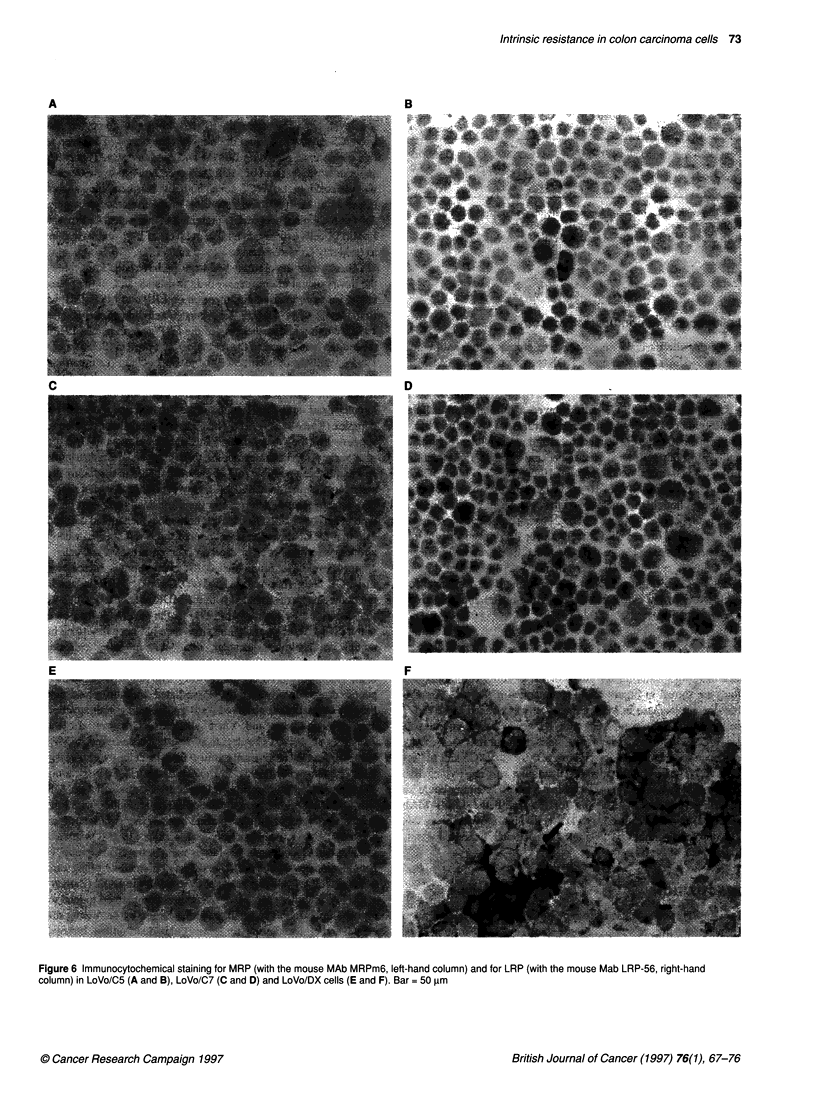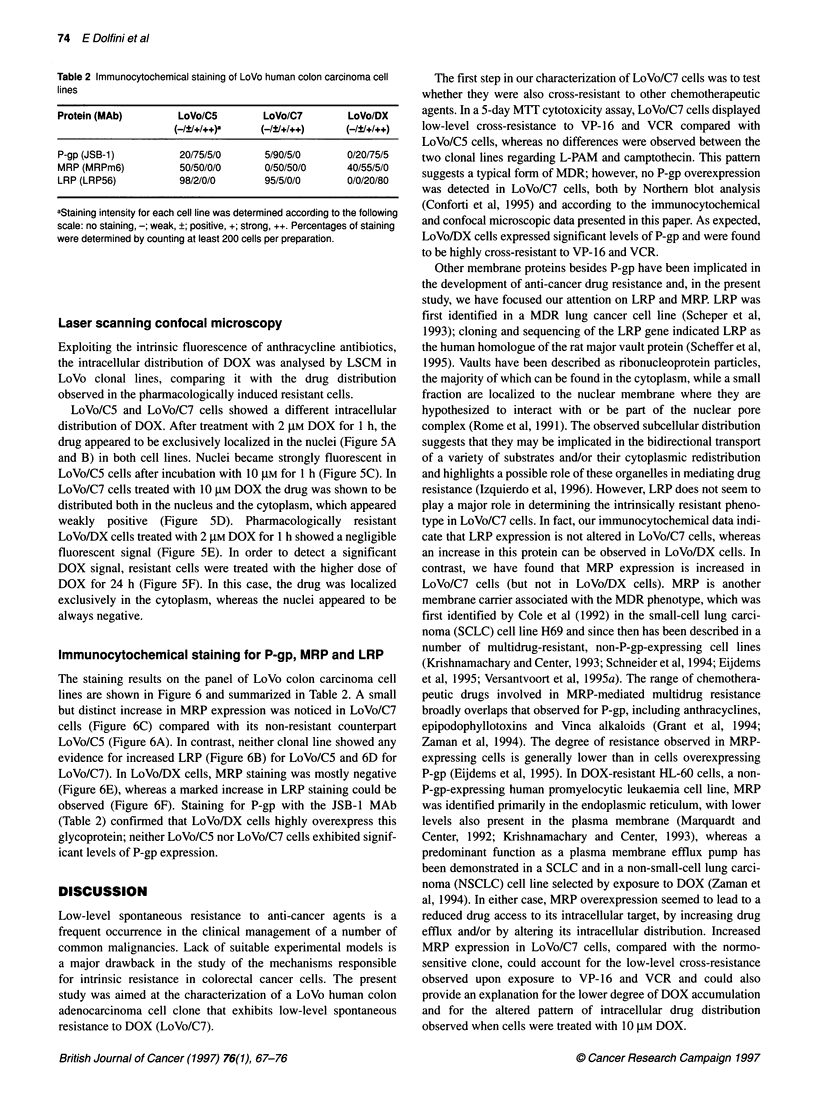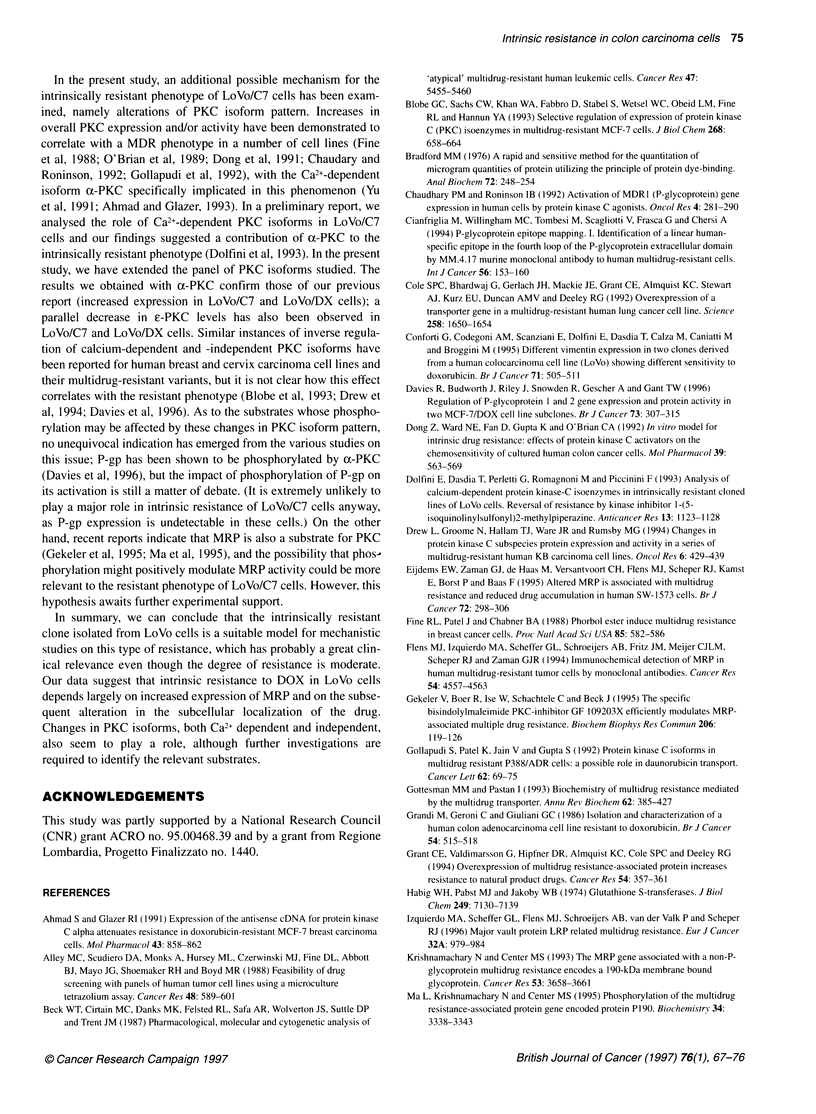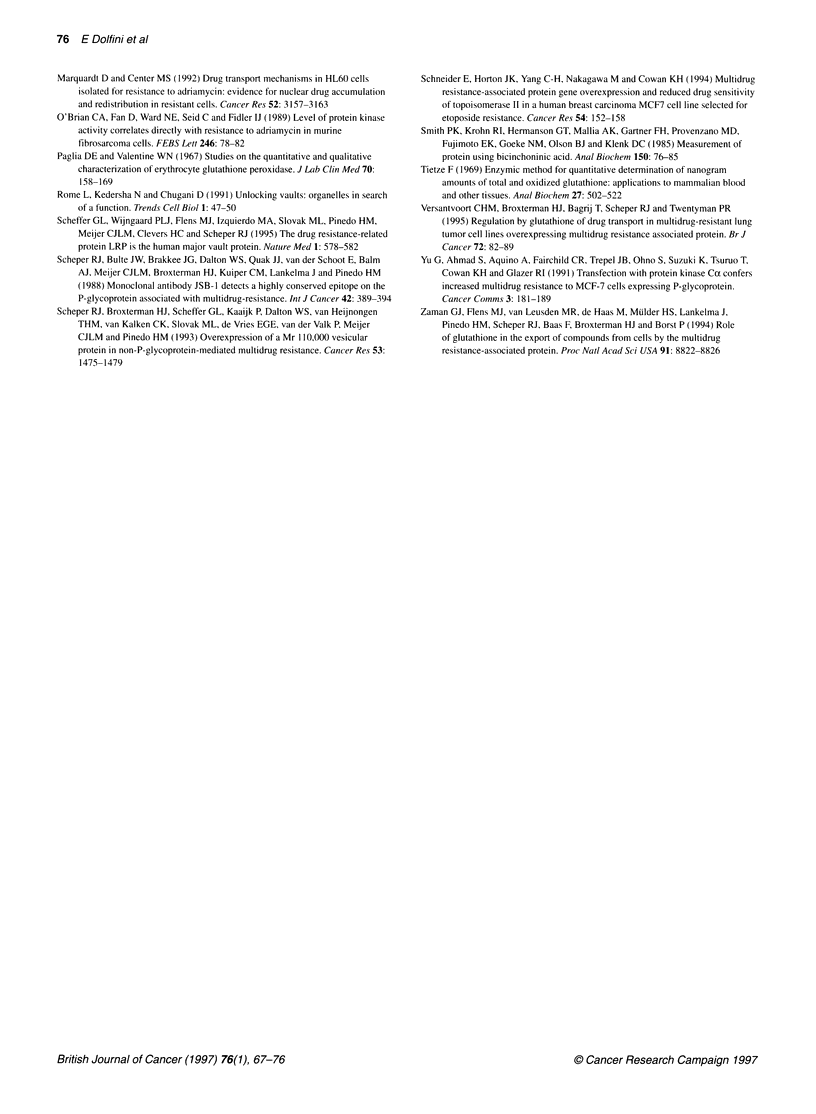# Characterization of a clonal human colon adenocarcinoma line intrinsically resistant to doxorubicin.

**DOI:** 10.1038/bjc.1997.338

**Published:** 1997

**Authors:** E. Dolfini, T. Dasdia, G. Arancia, A. Molinari, A. Calcabrini, R. J. Scheper, M. J. Flens, M. B. Gariboldi, E. Monti

**Affiliations:** Department of Biology and Genetics for Health Sciences, University of Milano, Milan, Italy.

## Abstract

**Images:**


British Journal of Cancer (1997) 76(1), 67–76
© 1997 Cancer Research Campaign

### Characterization of a clonal human colon adenocarcinoma line intrinsically resistant to doxorubicin

**E Dolfini[1], T Dasdia[1], G Arancia[2], A Molinari[2], A Calcabrini[2], RJ Scheper[3], MJ Flens[3], MB Gariboldi[4] and E Monti[4]**

[1]Department of Biology and Genetics for Health Sciences, University of Milano, Via Viotti, 3-I20133 Milan, Italy; [2]Department of Ultrastructure, Istituto Superiore di Sanità, Viale Regina Elena, 299-I00161 Rome, Italy; [3]Department of Pathology, Free University Hospital, 1081 HV Amsterdam, The Netherlands; [4]Institute of Pharmacology, University of Milano, Via Vanvitelli, 32-I20129 Milan, Italy

**Summary** Intrinsic low-level resistance to anti-cancer drugs is a major problem in the treatment of gastrointestinal malignancies. To address the problem presented by intrinsically resistant tumours, we have isolated two monoclonal lines from LoVo human colon adenocarcinoma cells: LoVo/C7, which is intrinsically resistant to doxorubicin (DOX); and LoVo/C5, which shows the same resistance index for DOX as the mixed parental cell population. For comparison, we have included in the study a LoVo-resistant line selected by continuous exposure to DOX and expressing a typical multidrug resistant (MDR) phenotype. In these cell lines we have studied the expression and/or activity of a number of proteins, including P-glycoprotein 170 (P-gp), multidrug resistance-associated protein (MRP), lung resistance-related protein (LRP), glutathione (GSH)-dependent enzymes and protein kinase C (PKC) isoforms, which have been implicated in anti-cancer drug resistance. Intracellular DOX distribution has been assessed by confocal microscopy. The results of the present study indicate that resistance in LoVo/C7 cells cannot be attributed to alterations in P-gp, LRP or GSH/GSH-dependent enzyme levels. Increased expression of MRP, accompanied by alterations in the subcellular distribution of DOX, has been observed in LoVo/C7 cells; changes in PKC isoform pattern have been detected in both intrinsically and pharmacologically resistant cells.

**Keywords:** intrinsic drug resistance, doxorubicin; colon adenocarcinoma; multidrug resistance-associated protein; lung resistance-related protein

The frequent occurrence of resistance to anti-cancer drugs is a major problem in the treatment of gastrointestinal malignancies. Two forms of drug resistance can be distinguished: tumour cells are initially insensitive to chemotherapy (intrinsic or de novo resistance) or they become insensitive after an initial response following selection by the agent used for treatment (acquired resistance). The multidrug resistant (MDR) phenotype, which is frequently observed in cultured tumour cells exposed to antineoplastic agents, is a typical example of acquired resistance, involving a lack of response to a host of chemotherapeutic agents and thereby drastically limiting the efficacy of pharmacological interventions. An impressive body of experimental evidence has accumulated over the past decade concerning the mechanisms underlying the development of MDR. Overexpression of P-glycoprotein 170 (P-gp), a membrane transporter for lipophilic xenobiotics, has been identified as a common occurrence in many MDR cell lines (Gottesman and Pastan, 1993), although a number of alternative or additional mechanisms have been described (Beck et al, 1987; Cole et al, 1992; Scheper et al, 1993). However, the MDR phenomenon largely depends on the experimental set-up, and it is still unclear how relevant it might be to the clinic. In contrast, very few studies have addressed the problem presented by intrinsically resistant tumours, which include some of the most frequently lethal malignancies (colorectal and lung cancers) (Dong et al, 1992). As a consequence, the mechanisms responsible for intrinsic drug resistance and its correlation with the MDR phenotype remain largely to be established.

A major stumbling block in in vitro resistance studies is the fact that most available tumour cell lines are in fact mixed populations, with heterogeneous genetic patterns and variable chemosensitivity. To overcome this difficulty, we have isolated a series of sublines from LoVo human colon adenocarcinoma cells and from this series we have selected two clones: LoVo/C7, which is intrinsically resistant to doxorubicin (DOX), and LoVo/C5, which exhibits the same degree of response to DOX as the mixed parental cell population (LoVo/WT). In these two clones we have studied, using biochemical and immunocytochemical techniques, the role played by a number of proteins, including P-gp, MRP (multidrug resistance-associated protein), LRP (lung resistance-related protein) and glutathione (GSH)-dependent enzymes, in determining the intrinsically resistant phenotype of LoVo/C7 cells. Intracellular DOX distribution has been assessed by confocal microscopy. In addition, based on preliminary results obtained on these cell lines (Dolfini et al, 1993), a detailed study of protein kinase C (PKC) isoforms was performed. For comparison, we have included in the study a LoVo-resistant line selected by continuous exposure to DOX and expressing a typical MDR phenotype (LoVo/DX) (Grandi et al, 1986). The results of the present study show that the resistant phenotype observed in LoVo/C7 cells cannot be attributed to alterations of P-glycoprotein, LRP or

*Received 8 August 1996*
*Revised 27 November 1996*
*Accepted 4 December 1996*

*Correspondence to:* E Monti, Institute of Pharmacology, Applied Pharmacology Section, University of Milano, Via Vanvitelli, 32-I20129 Milan, Italy

GSH/GSH-dependent enzyme levels. Increased expression of MRP, accompanied by alterations in the subcellular distribution of DOX, has been observed in LoVo/C7 cells; changes in PKC isoform pattern have been detected in both intrinsically and pharmacologically resistant cells.

### MATERIAL AND METHODS

### Cell lines

Two clones (LoVo/C5 and LoVo/C7) were isolated from the human wild-type LoVo colocarcinoma cell line as detailed elsewhere (Dolfini et al, 1993) and were subsequently grown in vitro in Ham's F12 medium supplemented with 10% fetal bovine serum and vitamins (Mascia Brunelli, Milan, Italy) and maintained at 37°C in a 5% carbon dioxide atmosphere. The LoVo/DX cells were obtained by exposure to increasing concentrations of doxorubicin and maintained under the same culture conditions (Grandi et al, 1986).

### Cytotoxicity studies

Growth inhibition by DOX, etoposide (VP-16), melphalan (L-PAM), vincristine (VCR) and camptothecin (CPT) was determined by the MTT assay (Alley et al, 1988). Approximately 3000–5000 cells per well were plated onto 96-well plates (Nunc, Denmark) and allowed to attach for 24 h before treatment with a range of drug concentrations for 5 days. A 5-day exposure time was chosen to allow the cells to grow in the presence of the drug for at least two doubling times. Doubling times ranged from 27 h 42 min for LoVo/WT to 57 h 15 min for LoVo/DX; initial cell densities were adjusted accordingly to obtain a 80–90% confluent monolayer in control wells at the end of the experiment. VP-16 and CPT stock solutions were prepared in dimethyl sulphoxide (DMSO) and subsequently diluted in complete tissue culture medium (final DMSO concentration $\leq$ 1% v/v); L-PAM was dissolved in a minimal volume of perchloric acid and then diluted in medium to the appropriate concentration.

### Enzyme assays

Subconfluent cell monolayers were harvested with EDTA, washed three times with phosphate-buffered saline (PBS), resuspended at $10^7$ cells $ml^{-1}$ in phosphate buffer and sonicated. The supernatants obtained by centrifugation of cell sonicates at 30 000 **g** were used to determine intracellular GSH levels and GSH-related enzyme activities. Selenium-dependent GSH peroxidase (GSHpx) activity was measured using the method described by Paglia and Valentine (1967), using 73 μM hydrogen peroxide as a substrate. Glutathione *S*-aryltransferase (GST) activity was determined by monitoring the formation of GSH-adducts with 1-chloro-2,4-dinitrobenzene (CDNB), according to the method described by Habig (1974). Protein concentration was measured using the Coomassie blue method as described by Bradford (1976). Total GSH levels were evaluated by the kinetic assay described by Tietze (1969) in perchloric acid-deproteinized samples. The data were analysed by means of the analysis of variance; multiple comparisons were evaluated by means of Scheffé's multiple range test (*P*-level 0.05).

**Table 1** Cytotoxic effects of different antineoplastic agents on LoVo human colon adenocarcinoma cell lines expressed as $IC_{50}$ values obtained in a 5-day MTT assay

| | LoVo/C5 (RI)[a] | LoVo/C7 (RI)[a] | LoVo/DX (RI)[a] |
|---|---|---|---|
| Doxorubicin | 20.1 nM (1) | 37.7 nM (1.88) | 1.3 μM (64.03) |
| Etoposide | 0.4 μM (1) | 0.5 μM (1.43) | 25.4 μM (71.06) |
| Melphalan | 9.0 μM (1) | 6.8 μM (0.76) | 3.6 μM (0.40) |
| Vincristine | 2.5 nM (1) | 4.8 nM (1.95) | 0.8 μM (321) |
| Camptothecin | 52.2 nM (1) | 45.8 nM (0.88) | 14.7 nM (0.28) |

[a]RI, resistance index relative to LoVo/C5.

### Western blot analysis of whole cell lysates

Cells were grown to subconfluence in 75-$cm^2$ flasks and trypsinized. Cell suspensions were washed in PBS and resuspended in 20 μl of lysis buffer (10 mM Tris, pH 7.5; 1 mM EDTA; 1 mM phenylmethylsulphonylfluoride; 10 μg $ml^{-1}$ leupeptin, 1% Triton X-100) per $10^6$ cells. Cells were then sonicated for 8 s and the material centrifuged in an Eppendorff microtube centrifuge at maximum speed for 20 min. After assessing the protein content using the BCA assay (Pierce Europe, The Netherlands) (Smith et al, 1985), supernatants were mixed with Laemmli sample buffer and boiled. Fifty micrograms of protein for each sample were loaded on a 10% polyacrylamide gel and electrophoresed. Proteins were transferred onto nitrocellulose membranes and incubated for 1 h with 5% non-fat dry milk. Blots were exposed to isoform-specific rabbit polyclonal antibodies (Boehringer Mannheim, Italy), diluted according to the manufacturer's instructions for 1 h and then washed in PBS. To visualize immunoreactive bands, a chemiluminescence kit (Boehringer Mannheim, Italy) was used, following the manufacturers' protocols.

### Flow cytometry

Time course accumulation studies were performed by flow cytometry on LoVo/C5, LoVo/C7 and LoVo/DX cells treated with DOX for up to 3 h. After treatment with 2 μM or 10 μM DOX, cells were washed with ice-cold Hank's balanced salt solution (HBSS), detached with EDTA and trypsin and resuspended in ice-cold PBS. For efflux studies, after washing with HBSS cells were incubated in drug-free medium at 37°C up to 4 h. They were then analysed on a FACScan flow cytometer (Becton Dickinson, Mountain View, CA, USA) equipped with a 15 mW, 488 nm, air-cooled argon ion laser. Fluorescence emission was collected after passing through a 585 nm bandpass filter.

For determination of surface expression of P-glycoprotein, cell suspensions were incubated for 30 min at 4°C with the monoclonal antibody MM.4.17 (10 μg $ml^{-1}$) (Cianfriglia et al, 1994). After washing with ice-cold PBS containing 10 mM sodium azide, 0.1% bouine serum albumin (BSA) and 0.02% EDTA, cells were incubated for 30 min at 4°C with 1:50 FITC-conjugated goat anti-mouse IgG (Sigma Chemical, St Louis, MO, USA); after several washings, cells were immediately analysed. Cells incubated with the second antibody only were used as negative controls.

### Laser scanning confocal microscopy (LSCM)

The intracellular distribution of DOX in different LoVo cell lines was studied by laser scanning confocal microscopy (LSCM). These studies were performed on living cells, grown on coverslips and mounted on glass microscope slides in the presence of the

© Cancer Research Campaign 1997

**Figure 1** Determination of glutathione levels (**A**), selenium-dependent glutathione peroxidase (**B**) and glutathione-*S*-transferase in LoVo cell lines. Values are the means ±s.d. of four independent experiments. *$P < 0.05$ vs LoVo/C5

growth medium. In order to avoid cell damage, image acquisitions were quickly performed on several cells per sample, acquiring signals coming from one field per coverslip. The observations were carried out with a Molecular Dynamics Sarastro 2000 CSLM (Molecular Dynamics, Sunnyvale, CA, USA) equipped with a 25-mW argon laser coupled to an epifluorescence Nikon Optiphot microscope with a 60x oil-immersion objective lens (NA=1.4). The 488-nm excitation filter, 510-nm primary dichroic beam-splitter and 510-nm detector filter were used. Images were acquired in average accumulation mode, with an image size of 1024x1024 pixels and pixel sizes of 0.08 μm. The optical sectioning was carried out with a 0.9-μm step size. Images were stored and processed with a Silicon Graphics Computer. In order to visualize both surface and internal structures, look-through projection processing was performed. This method allows the summarizing of voxel intensities along projection rays – the rays lie perpendicular to the plane of the sample. Acquisitions were recorded using the pseudocolour intensity representation.

### Immunocytochemical staining

P-gp expression was investigated by immunocytochemistry using the avidin–biotin complex method with the monoclonal antibody (MAb) JSB-1, which reacts against an internal epitope of P-gp (Scheper et al, 1988). For MRP expression, the mouse MAb MRPm6 (IgG1) was used. It was obtained after immunization with a fusion protein containing amino acids 1294–1430 plus 1497–1531 of the MRP protein (Flens et al, 1994). MRPm6 has been extensively characterized by protein blot analysis, immunocytochemical and immunohistochemical studies, and it does not cross-react with human P-gps (Flens et al, 1994). For LRP expression, the mouse MAb LRP-56 (IgG2b) was used. LRP-56 was raised by immunization with the non-P-gp MDR lung cancer cell line SW-1573/2R120, and it has been well characterized by immunoprecipitation and immunocytochemical analysis. LRP-56 specifically recognized a 110-kDa protein (LRP) overexpressed in a number of non-P-gp MDR cancer cell lines (Scheper et al, 1993; Scheffer et al, 1995). Immunocytochemistry was performed on unfixed cytospin preparations. Slides were incubated with normal rabbit serum for 15 min and then with MRPm6 1:10 or LRP-56 1:500 for 1 h. Rabbit anti-mouse biotin conjugate (1:150 for 30 min; Zymed Laboratories, San Francisco, CA, USA) and horseradish–streptavidin (1:500 for 1 h; Zymed) were the second and third steps respectively. Amino-ethylcarbazole (ICN Biochemicals, Aurora, OH USA) was used as a chromogen. Slides were counterstained with haematoxylin. Negative control slides were treated as above, substituting the primary antibody with an irrelevant IgG or PBS. GLC4S and GLC4/DOX cells, and SW-1573 and SW-1573/2R120 cells served as controls for MRP and LRP expression respectively (Scheper et al, 1993; Flens et al, 1994). Evaluation was done on coded slides to avoid bias in scoring cell lines.

### RESULTS

### Cytotoxicity studies

Table 1 shows the effects of DOX, VP-16, L-PAM, VCR and CPT expressed as the $IC_{50}$ values obtained after 5-day exposure to the drugs. LoVo/DX cells exhibited a marked cross-resistance to DOX, VP-16 and VCR, as expected from a P-gp expressing cell line; however, these cells showed a slight increase in their sensitivity to L-PAM and CPT compared with the two clonal lines.

### GSH and GSH-dependent enzymes

No significant differences in GSH levels (Figure 1A) and Selenium-dependent GSHpx activity (Figure 1B) were detected among the three LoVo cell lines tested. In contrast, a slight but significant decrease in GST activity was observed in LoVo/C7 cells compared with the two other cell lines, which had similar values for this enzyme (Figure 1C).

© Cancer Research Campaign 1997

**Figure 2** Western blot analysis of PKC isoforms in LoVo cells

### Analysis of PKC isoforms

Of the tested PKC isoenzymes, α-, δ-, ε- and ζ-isoforms were detectable in the three cell lines, although some differences in expression were apparent. In fact, while δ- and ζ-PKC protein levels were similar in all cell lines, marked differences were observed in α- and ε-PKC expression: higher levels of the α-isoform were found in both LoVo/DX and LoVo/C7 cells compared with LoVo/C5 cells, whereas ε-PKC was markedly decreased in both resistant lines, particularly in LoVo/C7 cells (Figure 2). In contrast, β-PKC was found to be barely detectable in these cell lines by a preliminary test and therefore was not analysed further.

### Flow cytometry

Flow cytometric determination of surface P-glycoprotein expression showed that only LoVo/DX cells were positive for MM.4.17 labelling, while the clonal lines LoVo/C5 and LoVo/C7 did not show any reactivity with this antibody (Figure 3)

Figure 4 shows the time course of drug accumulation (up to 3 h) and efflux (up to 4 h) in cells treated with 2 μM and 10 μM DOX (Figure 4A and B respectively). When the lower dose was used, no significant difference in intracellular fluorescence was observed between LoVo/C5 and LoVo/C7 cells; however, when cells were treated with 10 μM DOX, LoVo/C5 cells seemed to accumulate a higher drug amount than LoVo/C7. In any case, a very low drug uptake was detected in LoVo/DX cells. When the cells were allowed to recover in drug-free medium for 4 h LoVo/DX cells showed a complete efflux of the drug, while in the other lines the efficiency of extrusion of the drug was much lower. However, a small difference in efflux rate can be observed between LoVo/C5 and LoVo/C7 cells, efflux being significantly faster in the latter.

**Figure 3** Flow cytometric analysis of cell surface P-glycoprotein is indicated by the fluorescence intensity (on the abscissa) of the antibodies bound to the cells. The MDR variants of LoVo cells (LoVo/DX) appeared to be positive for Mab MM4.17 (right curve in **A**). The two clones LoVo/C5 and LoVo/C7 (**B** and **C**) did not show significant reactivity against this antibody

© Cancer Research Campaign 1997

**Figure 4** Flow cytometric analysis of the time course of doxorubicin accumulation and efflux in LoVo cells treated with doxorubicin 2 μM (**A**) or 10 μM (**B**) for 3 h and then recovered in drug-free medium for 4 h. Each data point is the mean ± s.e. of three independent experiments. ○, LoVo/C5; ●, LoVo/C7; ◇, LoVo/DX

**Figure 5** Intracellular distribution of DOX in living LoVo cells detected by laser scanning confocal microscopic determination of (**A**) LoVo/C5 and (**B**) LoVo/C7 treated with 2 μM DOX for 1 h, (**C**) LoVo/C5 and (**D**) LoVo/C7 cells treated with 10 μM DOX for 1 h, (**E** and **F**) LoVo/DX cells treated with 2 μM DOX for 1 h and 10 μM DOX for 24 h respectively. Bar = 30 μm

© Cancer Research Campaign 1997

**Figure 6** Immunocytochemical staining for MRP (with the mouse MAb MRPm6, left-hand column) and for LRP (with the mouse Mab LRP-56, right-hand column) in LoVo/C5 (**A** and **B**), LoVo/C7 (**C** and **D**) and LoVo/DX cells (**E** and **F**). Bar = 50 µm

Table 2 Immunocytochemical staining of LoVo human colon carcinoma cell lines

| Protein (MAb) | LoVo/C5 (–/±/+/++)[a] | LoVo/C7 (–/±/+/++) | LoVo/DX (–/±/+/++) |
|---|---|---|---|
| P-gp (JSB-1) | 20/75/5/0 | 5/90/5/0 | 0/20/75/5 |
| MRP (MRPm6) | 50/50/0/0 | 0/50/50/0 | 40/55/5/0 |
| LRP (LRP56) | 98/2/0/0 | 95/5/0/0 | 0/0/20/80 |

[a]Staining intensity for each cell line was determined according to the following scale: no staining, –; weak, ±; positive, +; strong, ++. Percentages of staining were determined by counting at least 200 cells per preparation.

### Laser scanning confocal microscopy

Exploiting the intrinsic fluorescence of anthracycline antibiotics, the intracellular distribution of DOX was analysed by LSCM in LoVo clonal lines, comparing it with the drug distribution observed in the pharmacologically induced resistant cells.

LoVo/C5 and LoVo/C7 cells showed a different intracellular distribution of DOX. After treatment with 2 μM DOX for 1 h, the drug appeared to be exclusively localized in the nuclei (Figure 5A and B) in both cell lines. Nuclei became strongly fluorescent in LoVo/C5 cells after incubation with 10 μM for 1 h (Figure 5C). In LoVo/C7 cells treated with 10 μM DOX the drug was shown to be distributed both in the nucleus and the cytoplasm, which appeared weakly positive (Figure 5D). Pharmacologically resistant LoVo/DX cells treated with 2 μM DOX for 1 h showed a negligible fluorescent signal (Figure 5E). In order to detect a significant DOX signal, resistant cells were treated with the higher dose of DOX for 24 h (Figure 5F). In this case, the drug was localized exclusively in the cytoplasm, whereas the nuclei appeared to be always negative.

### Immunocytochemical staining for P-gp, MRP and LRP

The staining results on the panel of LoVo colon carcinoma cell lines are shown in Figure 6 and summarized in Table 2. A small but distinct increase in MRP expression was noticed in LoVo/C7 cells (Figure 6C) compared with its non-resistant counterpart LoVo/C5 (Figure 6A). In contrast, neither clonal line showed any evidence for increased LRP (Figure 6B) for LoVo/C5 and 6D for LoVo/C7). In LoVo/DX cells, MRP staining was mostly negative (Figure 6E), whereas a marked increase in LRP staining could be observed (Figure 6F). Staining for P-gp with the JSB-1 MAb (Table 2) confirmed that LoVo/DX cells highly overexpress this glycoprotein; neither LoVo/C5 nor LoVo/C7 cells exhibited significant levels of P-gp expression.

### DISCUSSION

Low-level spontaneous resistance to anti-cancer agents is a frequent occurrence in the clinical management of a number of common malignancies. Lack of suitable experimental models is a major drawback in the study of the mechanisms responsible for intrinsic resistance in colorectal cancer cells. The present study was aimed at the characterization of a LoVo human colon adenocarcinoma cell clone that exhibits low-level spontaneous resistance to DOX (LoVo/C7).

The first step in our characterization of LoVo/C7 cells was to test whether they were also cross-resistant to other chemotherapeutic agents. In a 5-day MTT cytotoxicity assay, LoVo/C7 cells displayed low-level cross-resistance to VP-16 and VCR compared with LoVo/C5 cells, whereas no differences were observed between the two clonal lines regarding L-PAM and camptothecin. This pattern suggests a typical form of MDR; however, no P-gp overexpression was detected in LoVo/C7 cells, both by Northern blot analysis (Conforti et al, 1995) and according to the immunocytochemical and confocal microscopic data presented in this paper. As expected, LoVo/DX cells expressed significant levels of P-gp and were found to be highly cross-resistant to VP-16 and VCR.

Other membrane proteins besides P-gp have been implicated in the development of anti-cancer drug resistance and, in the present study, we have focused our attention on LRP and MRP. LRP was first identified in a MDR lung cancer cell line (Scheper et al, 1993); cloning and sequencing of the LRP gene indicated LRP as the human homologue of the rat major vault protein (Scheffer et al, 1995). Vaults have been described as ribonucleoprotein particles, the majority of which can be found in the cytoplasm, while a small fraction are localized to the nuclear membrane where they are hypothesized to interact with or be part of the nuclear pore complex (Rome et al, 1991). The observed subcellular distribution suggests that they may be implicated in the bidirectional transport of a variety of substrates and/or their cytoplasmic redistribution and highlights a possible role of these organelles in mediating drug resistance (Izquierdo et al, 1996). However, LRP does not seem to play a major role in determining the intrinsically resistant phenotype in LoVo/C7 cells. In fact, our immunocytochemical data indicate that LRP expression is not altered in LoVo/C7 cells, whereas an increase in this protein can be observed in LoVo/DX cells. In contrast, we have found that MRP expression is increased in LoVo/C7 cells (but not in LoVo/DX cells). MRP is another membrane carrier associated with the MDR phenotype, which was first identified by Cole et al (1992) in the small-cell lung carcinoma (SCLC) cell line H69 and since then has been described in a number of multidrug-resistant, non-P-gp-expressing cell lines (Krishnamachary and Center, 1993; Schneider et al, 1994; Eijdems et al, 1995; Versantvoort et al, 1995*a*). The range of chemotherapeutic drugs involved in MRP-mediated multidrug resistance broadly overlaps that observed for P-gp, including anthracyclines, epipodophyllotoxins and Vinca alkaloids (Grant et al, 1994; Zaman et al, 1994). The degree of resistance observed in MRP-expressing cells is generally lower than in cells overexpressing P-gp (Eijdems et al, 1995). In DOX-resistant HL-60 cells, a non-P-gp-expressing human promyelocytic leukaemia cell line, MRP was identified primarily in the endoplasmic reticulum, with lower levels also present in the plasma membrane (Marquardt and Center, 1992; Krishnamachary and Center, 1993), whereas a predominant function as a plasma membrane efflux pump has been demonstrated in a SCLC and in a non-small-cell lung carcinoma (NSCLC) cell line selected by exposure to DOX (Zaman et al, 1994). In either case, MRP overexpression seemed to lead to a reduced drug access to its intracellular target, by increasing drug efflux and/or by altering its intracellular distribution. Increased MRP expression in LoVo/C7 cells, compared with the normosensitive clone, could account for the low-level cross-resistance observed upon exposure to VP-16 and VCR and could also provide an explanation for the lower degree of DOX accumulation and for the altered pattern of intracellular drug distribution observed when cells were treated with 10 μM DOX.

© Cancer Research Campaign 1997

In the present study, an additional possible mechanism for the intrinsically resistant phenotype of LoVo/C7 cells has been examined, namely alterations of PKC isoform pattern. Increases in overall PKC expression and/or activity have been demonstrated to correlate with a MDR phenotype in a number of cell lines (Fine et al, 1988; O'Brian et al, 1989; Dong et al, 1991; Chaudary and Roninson, 1992; Gollapudi et al, 1992), with the $Ca^{2+}$-dependent isoform α-PKC specifically implicated in this phenomenon (Yu et al, 1991; Ahmad and Glazer, 1993). In a preliminary report, we analysed the role of $Ca^{2+}$-dependent PKC isoforms in LoVo/C7 cells and our findings suggested a contribution of α-PKC to the intrinsically resistant phenotype (Dolfini et al, 1993). In the present study, we have extended the panel of PKC isoforms studied. The results we obtained with α-PKC confirm those of our previous report (increased expression in LoVo/C7 and LoVo/DX cells); a parallel decrease in ε-PKC levels has also been observed in LoVo/C7 and LoVo/DX cells. Similar instances of inverse regulation of calcium-dependent and -independent PKC isoforms have been reported for human breast and cervix carcinoma cell lines and their multidrug-resistant variants, but it is not clear how this effect correlates with the resistant phenotype (Blobe et al, 1993; Drew et al, 1994; Davies et al, 1996). As to the substrates whose phosphorylation may be affected by these changes in PKC isoform pattern, no unequivocal indication has emerged from the various studies on this issue; P-gp has been shown to be phosphorylated by α-PKC (Davies et al, 1996), but the impact of phosphorylation of P-gp on its activation is still a matter of debate. (It is extremely unlikely to play a major role in intrinsic resistance of LoVo/C7 cells anyway, as P-gp expression is undetectable in these cells.) On the other hand, recent reports indicate that MRP is also a substrate for PKC (Gekeler et al, 1995; Ma et al, 1995), and the possibility that phosphorylation might positively modulate MRP activity could be more relevant to the resistant phenotype of LoVo/C7 cells. However, this hypothesis awaits further experimental support.

In summary, we can conclude that the intrinsically resistant clone isolated from LoVo cells is a suitable model for mechanistic studies on this type of resistance, which has probably a great clinical relevance even though the degree of resistance is moderate. Our data suggest that intrinsic resistance to DOX in LoVo cells depends largely on increased expression of MRP and on the subsequent alteration in the subcellular localization of the drug. Changes in PKC isoforms, both $Ca^{2+}$ dependent and independent, also seem to play a role, although further investigations are required to identify the relevant substrates.

### ACKNOWLEDGEMENTS

This study was partly supported by a National Research Council (CNR) grant ACRO no. 95.00468.39 and by a grant from Regione Lombardia, Progetto Finalizzato no. 1440.

### REFERENCES

Ahmad S and Glazer RI (1991) Expression of the antisense cDNA for protein kinase C alpha attenuates resistance in doxorubicin-resistant MCF-7 breast carcinoma cells. *Mol Pharmacol* **43**: 858–862

Alley MC, Scudiero DA, Monks A, Hursey ML, Czerwinski MJ, Fine DL, Abbott BJ, Mayo JG, Shoemaker RH and Boyd MR (1988) Feasibility of drug screening with panels of human tumor cell lines using a microculture tetrazolium assay. *Cancer Res* **48**: 589–601

Beck WT, Cirtain MC, Danks MK, Felsted RL, Safa AR, Wolverton JS, Suttle DP and Trent JM (1987) Pharmacological, molecular and cytogenetic analysis of 'atypical' multidrug-resistant human leukemic cells. *Cancer Res* **47**: 5455–5460

Blobe GC, Sachs CW, Khan WA, Fabbro D, Stabel S, Wetsel WC, Obeid LM, Fine RL and Hannun YA (1993) Selective regulation of expression of protein kinase C (PKC) isoenzymes in multidrug-resistant MCF-7 cells. *J Biol Chem* **268**: 658–664

Bradford MM (1976) A rapid and sensitive method for the quantitation of microgram quantities of protein utilizing the principle of protein dye-binding. *Anal Biochem* **72**: 248–254

Chaudhary PM and Roninson IB (1992) Activation of MDR1 (P-glycoprotein) gene expression in human cells by protein kinase C agonists. *Oncol Res* **4**: 281–290

Cianfriglia M, Willingham MC, Tombesi M, Scagliotti V, Frasca G and Chersi A (1994) P-glycoprotein epitope mapping. I. Identification of a linear human-specific epitope in the fourth loop of the P-glycoprotein extracellular domain by MM.4.17 murine monoclonal antibody to human multidrug-resistant cells. *Int J Cancer* **56**: 153–160

Cole SPC, Bhardwaj G, Gerlach JH, Mackie JE, Grant CE, Almquist KC, Stewart AJ, Kurz EU, Duncan AMV and Deeley RG (1992) Overexpression of a transporter gene in a multidrug-resistant human lung cancer cell line. *Science* **258**: 1650–1654

Conforti G, Codegoni AM, Scanziani E, Dolfini E, Dasdia T, Calza M, Caniatti M and Broggini M (1995) Different vimentin expression in two clones derived from a human colocarcinoma cell line (LoVo) showing different sensitivity to doxorubicin. *Br J Cancer* **71**: 505–511

Davies R, Budworth J, Riley J, Snowden R, Gescher A and Gant TW (1996) Regulation of P-glycoprotein 1 and 2 gene expression and protein activity in two MCF-7/DOX cell line subclones. *Br J Cancer* **73**: 307–315

Dong Z, Ward NE, Fan D, Gupta K and O'Brian CA (1992) *In vitro* model for intrinsic drug resistance: effects of protein kinase C activators on the chemosensitivity of cultured human colon cancer cells. *Mol Pharmacol* **39**: 563–569

Dolfini E, Dasdia T, Perletti G, Romagnoni M and Piccinini F (1993) Analysis of calcium-dependent protein kinase-C isoenzymes in intrinsically resistant cloned lines of LoVo cells. Reversal of resistance by kinase inhibitor 1-(5-isoquinolinylsulfonyl)2-methylpiperazine. *Anticancer Res* **13**: 1123–1128

Drew L, Groome N, Hallam TJ, Ware JR and Rumsby MG (1994) Changes in protein kinase C subspecies protein expression and activity in a series of multidrug-resistant human KB carcinoma cell lines. *Oncol Res* **6**: 429–439

Eijdems EW, Zaman GJ, de Haas M, Versantvoort CH, Flens MJ, Scheper RJ, Kamst E, Borst P and Baas F (1995) Altered MRP is associated with multidrug resistance and reduced drug accumulation in human SW-1573 cells. *Br J Cancer* **72**: 298–306

Fine RL, Patel J and Chabner BA (1988) Phorbol ester induce multidrug resistance in breast cancer cells. *Proc Natl Acad Sci USA* **85**: 582–586

Flens MJ, Izquierdo MA, Scheffer GL, Schroeijers AB, Fritz JM, Meijer CJLM, Scheper RJ and Zaman GJR (1994) Immunochemical detection of MRP in human multidrug-resistant tumor cells by monoclonal antibodies. *Cancer Res* **54**: 4557–4563

Gekeler V, Boer R, Ise W, Schachtele C and Beck J (1995) The specific bisindolylmaleimide PKC-inhibitor GF 109203X efficiently modulates MRP-associated multiple drug resistance. *Biochem Biophys Res Commun* **206**: 119–126

Gollapudi S, Patel K, Jain V and Gupta S (1992) Protein kinase C isoforms in multidrug resistant P388/ADR cells: a possible role in daunorubicin transport. *Cancer Lett* **62**: 69–75

Gottesman MM and Pastan I (1993) Biochemistry of multidrug resistance mediated by the multidrug transporter. *Annu Rev Biochem* **62**: 385–427

Grandi M, Geroni C and Giuliani GC (1986) Isolation and characterization of a human colon adenocarcinoma cell line resistant to doxorubicin. *Br J Cancer* **54**: 515–518

Grant CE, Valdimarsson G, Hipfner DR, Almquist KC, Cole SPC and Deeley RG (1994) Overexpression of multidrug resistance-associated protein increases resistance to natural product drugs. *Cancer Res* **54**: 357–361

Habig WH, Pabst MJ and Jakoby WB (1974) Glutathione S-transferases. *J Biol Chem* **249**: 7130–7139

Izquierdo MA, Scheffer GL, Flens MJ, Schroeijers AB, van der Valk P and Scheper RJ (1996) Major vault protein LRP related multidrug resistance. *Eur J Cancer* **32A**: 979–984

Krishnamachary N and Center MS (1993) The MRP gene associated with a non-P-glycoprotein multidrug resistance encodes a 190-kDa membrane bound glycoprotein. *Cancer Res* **53**: 3658–3661

Ma L, Krishnamachary N and Center MS (1995) Phosphorylation of the multidrug resistance-associated protein gene encoded protein P190. *Biochemistry* **34**: 3338–3343

© Cancer Research Campaign 1997

Marquardt D and Center MS (1992) Drug transport mechanisms in HL60 cells isolated for resistance to adriamycin: evidence for nuclear drug accumulation and redistribution in resistant cells. *Cancer Res* **52**: 3157–3163

O'Brian CA, Fan D, Ward NE, Seid C and Fidler IJ (1989) Level of protein kinase activity correlates directly with resistance to adriamycin in murine fibrosarcoma cells. *FEBS Lett* **246**: 78–82

Paglia DE and Valentine WN (1967) Studies on the quantitative and qualitative characterization of erythrocyte glutathione peroxidase. *J Lab Clin Med* **70**: 158–169

Rome L, Kedersha N and Chugani D (1991) Unlocking vaults: organelles in search of a function. *Trends Cell Biol* **1**: 47–50

Scheffer GL, Wijngaard PLJ, Flens MJ, Izquierdo MA, Slovak ML, Pinedo HM, Meijer CJLM, Clevers HC and Scheper RJ (1995) The drug resistance-related protein LRP is the human major vault protein. *Nature Med* **1**: 578–582

Scheper RJ, Bulte JW, Brakkee JG, Dalton WS, Quak JJ, van der Schoot E, Balm AJ, Meijer CJLM, Broxterman HJ, Kuiper CM, Lankelma J and Pinedo HM (1988) Monoclonal antibody JSB-1 detects a highly conserved epitope on the P-glycoprotein associated with multidrug-resistance. *Int J Cancer* **42**: 389–394

Scheper RJ, Broxterman HJ, Scheffer GL, Kaaijk P, Dalton WS, van Heijnongen THM, van Kalken CK, Slovak ML, de Vries EGE, van der Valk P, Meijer CJLM and Pinedo HM (1993) Overexpression of a Mr 110,000 vesicular protein in non-P-glycoprotein-mediated multidrug resistance. *Cancer Res* **53**: 1475–1479

Schneider E, Horton JK, Yang C-H, Nakagawa M and Cowan KH (1994) Multidrug resistance-associated protein gene overexpression and reduced drug sensitivity of topoisomerase II in a human breast carcinoma MCF7 cell line selected for etoposide resistance. *Cancer Res* **54**: 152–158

Smith PK, Krohn RI, Hermanson GT, Mallia AK, Gartner FH, Provenzano MD, Fujimoto EK, Goeke NM, Olson BJ and Klenk DC (1985) Measurement of protein using bicinchoninic acid. *Anal Biochem* **150**: 76–85

Tietze F (1969) Enzymic method for quantitative determination of nanogram amounts of total and oxidized glutathione: applications to mammalian blood and other tissues. *Anal Biochem* **27**: 502–522

Versantvoort CHM, Broxterman HJ, Bagrij T, Scheper RJ and Twentyman PR (1995) Regulation by glutathione of drug transport in multidrug-resistant lung tumor cell lines overexpressing multidrug resistance associated protein. *Br J Cancer* **72**: 82–89

Yu G, Ahmad S, Aquino A, Fairchild CR, Trepel JB, Ohno S, Suzuki K, Tsuruo T, Cowan KH and Glazer RI (1991) Transfection with protein kinase Cα confers increased multidrug resistance to MCF-7 cells expressing P-glycoprotein. *Cancer Comms* **3**: 181–189

Zaman GJ, Flens MJ, van Leusden MR, de Haas M, Mülder HS, Lankelma J, Pinedo HM, Scheper RJ, Baas F, Broxterman HJ and Borst P (1994) Role of glutathione in the export of compounds from cells by the multidrug resistance-associated protein. *Proc Natl Acad Sci USA* **91**: 8822–8826

*© Cancer Research Campaign 1997*